# Identification of key genes for hypertrophic cardiomyopathy using integrated network analysis of differential lncRNA and gene expression

**DOI:** 10.3389/fcvm.2022.946229

**Published:** 2022-08-04

**Authors:** Jing Cao, Lei Yuan

**Affiliations:** ^1^Department of Cardiovascular Medicine, Third Xiangya Hospital, Central South University, Changsha, China; ^2^Department of Medical Affairs, Xiangya Hospital, Central South University, Changsha, China

**Keywords:** hypertrophic cardiomyopathy, differentially expressed long non-coding RNAs, differentially expressed mRNAs, co-expression network, bioinformatics analysis

## Abstract

**Objective:**

Hypertrophic cardiomyopathy (HCM) is a complex heterogeneous heart disease. Recent reports found that long non-coding RNAs (lncRNAs) play an important role in the progression of cardiovascular diseases. The present study aimed to identify the novel lncRNAs and messenger RNAs (mRNAs) and determine the key pathways involved in HCM.

**Methods:**

The lncRNA and mRNA sequencing datasets of GSE68316 and GSE130036 were downloaded from the Gene Expression Omnibus (GEO) database. An integrated co-expression network analysis was conducted to identify differentially expressed lncRNAs and differentially expressed mRNAs in patients with HCM. Then, gene ontology (GO) and Kyoto Encyclopedia of Genes and Genomes (KEGG) pathway enrichment analyses were explored to identify the biological functions and signaling pathways of the co-expression network. Protein–protein interaction (PPI) and hub gene networks were constructed by using Cytoscape software. Plasma samples of patients with HCM and the GSE89714 dataset were used to validate the bioinformatics results.

**Results:**

A total of 1,426 differentially expressed long non-coding RNAs (lncRNAs) and 1,715 differentially expressed mRNAs were obtained from GSE68316, of which 965 lncRNAs and 896 mRNAs were upregulated and 461 lncRNAs and 819 mRNAs were downregulated. A total of 469 differentially expressed lncRNAs and 2,407 differentially expressed mRNAs were screened from GSE130036, of which 183 lncRNAs and 1,283 mRNAs were upregulated and 286 lncRNAs and 1,124 mRNAs were downregulated. A co-expression network was constructed and contained 30 differentially expressed lncRNAs and 63 differentially expressed mRNAs, which were primarily involved in ‘G-protein beta/gamma-subunit complex binding,' ‘polyubiquitin modification-dependent protein binding,' ‘Apelin signaling pathway,' and ‘Wnt signaling pathway.' The 10 hub genes in the upregulated network [G Protein Subunit Alpha I2 (GNAI2), G Protein Subunit Alpha I1 (GNAI1), G Protein Subunit Alpha I3 (GNAI3), G Protein Subunit Gamma 2 (GNG2), G Protein Subunit Beta 1 (GNB1), G Protein Subunit Gamma 13 (GNG13), G Protein Subunit Gamma Transducin 1 (GNGT1), G Protein Subunit Gamma 12 (GNG12), AKT Serine/Threonine Kinase 1 (AKT1) and GNAS Complex Locus (GNAS)] and the 10 hub genes in the downregulated network [Nucleotide-Binding Oligomerization Domain Containing Protein 2 (NOD2), Receptor-Interacting Serine/Threonine Kinase 2 (RIPK2), Nucleotide-Binding Oligomerization Domain Containing Protein 1 (NOD1), Mitochondrial Antiviral Signaling Protein (MAVS), Autophagy Related 16-Like 1 (ATG16L1), Interferon Induced With Helicase C Domain 1 (IFIH1), Autophagy Related 5 (ATG5), TANK-Binding Kinase 1 (TBK1), Caspase Recruitment Domain Family Member 9 (CARD9), and von Willebrand factor (VWF)] were screened using cytoHubba. The expression of LA16c-312E8.2 and RP5-1160K1.3 in the plasma of patients with HCM was elevated, and the expression of the MIR22 host gene (MIR22HG) was decreased, which was consistent with our analysis, while the expression of LINC00324 and Small Nucleolar RNA Host Gene 12 (SNHG12) was not significantly different between the two groups. Verification analyses performed on GSE89714 showed the upregulated mRNAs of Chloride Voltage-Gated Channel 7 (CLCN7), N-Acetylglucosamine-1-Phosphate Transferase Subunit Gamma (GNPTG), Unk Like Zinc Finger (UNKL), Adenosine Monophosphate Deaminase 2 (AMPD2), GNAI3, WD Repeat Domain 81 (WDR81), and Serpin Family F Member 1 (SERPINF1) and downregulated mRNAs of TATA-Box Binding Protein Associated Factor 12 (TAF12) co-expressed with five crucial lncRNAs. Moreover, GNAI2, GNAI3, GNG12, and vWF were upregulated and GNAS was downregulated in the top 10 hub genes of upregulated and downregulated PPI networks.

**Conclusion:**

These findings from integrative biological analysis of lncRNA-mRNA co-expression networks explored the key genes and pathways and provide new insights into the understanding of the mechanism and discovering new therapeutic targets for HCM. Three differentially expressed pivotal lncRNAs (LA16c-312E8.2, RP5-1160K1.3, and MIR22HG) in the co-expression network may serve as biomarkers and intervention targets for the diagnosis and treatment of HCM.

## Introduction

Hypertrophic cardiomyopathy (HCM) is a complex heterogeneous heart disease that has been recognized to be a significant cause of atrial fibrillation, heart failure, and arrhythmic sudden death and is one of the main causes of sudden cardiac death in young adults (1). Epidemiological investigations have shown that the prevalence of phenotypic expression of HCM in the adult general population was about 0.2% (1:500) (2, 3). HCM is characterized by myocardial hypertrophy, asymmetrical ventricular septal hypertrophy, ventricular narrowing, and abnormal myocardial cell hypertrophy. In the absence of other cardiac or systemic diseases, such as hypertension or aortic stenosis, echocardiography of a hypertrophic but undilated left ventricle is the easiest and most reliable method for the clinical diagnosis of HCM (4). The pathogenesis of HCM is strongly associated with the mutation of the genes encoding proteins of thick and thin myofilament contractile components of the cardiac sarcomere or Z-disk (5, 6). Although myosin heavy chain 7 (MYH7) and myosin binding protein C3 (MYBPC3), which encode β-myosin heavy chain and myosin binding protein C, are the two most common mutations (7, 8), the great heterogeneity and diversity in the molecular pathways make the exact mechanism of HCM remain indistinct.

About 99% of genomic sequences in the human genome do not encode proteins, but they are highly transcriptional and produce a broad spectrum of non-coding RNAs (ncRNAs) that show regulatory and structural functions. The encyclopedia of DNA elements (ENCODE) project and other studies showed that limiting the pathogenesis analysis of diseases to the protein-coding regions of the human genome is insufficient since many non-coding variations are associated with important human diseases (9–11). The most studied ncRNAs in the heart are microRNAs, and little is known about the other ncRNAs (12–14). Long non-coding RNAs (lncRNAs) are defined as RNAs with transcripts >200 nucleotides that do not encode protein. Recent reports demonstrated that lncRNAs play an important role in the progression of several cardiovascular diseases, such as acute myocardial infarction, heart failure, and atrial fibrillation (15–17). In HCM, lncRNAs are verified to be involved in vital biological processes of myocardial fibrosis, myocardial hypertrophy, and atherosclerosis (18, 19). For example, the overexpression of cardiomyocyte-specific non-coding repressor of factor of Nuclear factor of activated T-cells (NFAT) (NRON) exacerbated transverse aortic constriction (TAC)-induced hypertrophy in mice heart (19), and ROR sponges miR-133 to cause the re-expression of atrial natriuretic peptide and B-type natriuretic peptide, leading to the exacerbation of cardiac hypertrophy eventually (20). However, the role of lncRNAs in the progression of HCM remains to be further explored.

Currently, big data mining and precision medicine have gained considerable attention. In this study, we aimed to perform in-depth data mining based on former microarray studies. We identified differentially expressed lncRNAs and mRNAs in HCM progression by a comprehensive analysis of the public datasets GSE68316 and GSE13036, including 35 patients with HCM and 14 controls. Subsequently, we constructed the co-expression network of lncRNA-mRNA and performed gene ontology (GO) and Kyoto Encyclopedia of Genes/Genomes (KEGG) pathway enrichment analyses on the differentially expressed genes in the network. Finally, we constructed the protein–protein interaction (PPI) network of the differentially expressed genes to reveal the potential role of HCM-related mRNAs and lncRNAs. This study will provide useful information to explore the potential candidate biomarkers for HCM diagnosis, prognosis, and intervention targets.

## Materials and methods

### Data of gene expression profiles

The expression profile data of LncRNA and mRNA with the sequence numbers GSE68316 and GSE130036 were obtained from the Gene Expression Omnibus (GEO, http://www.ncbi.nlm.nih.gov/geo/) database. The left ventricular tissues of 35 patients with HCM and 14 healthy donors along with their clinical data were obtained, including 7 HCM and 5 healthy donors from GSE68316 and 28 HCM and 9 healthy donors from GSE130036. Gene expression profile dataset GSE89714 was collected from the GEO database to verify the key genes involved in HCM, which included the left ventricular tissues of 5 patients with HCM and 4 healthy donors. The characteristics of these expression profiles are presented in Supplementary Table 1.

### Screening of differentially expressed lncRNAs and mRNAs

R language was used to analyze the initial data and identify differential lncRNAs and mRNAs. The Affy package was applied to perform data normalization, including converting the data to raw data and correcting the background. The limma software package was performed to screen the data for differentially expressed lncRNAs and mRNAs. The thresholds were |log2 (fold change) |>1 with the adjusted *p*-value < 0.05, and the genes that met the criteria were considered as differentially expressed lncRNAs and mRNAs. The gglot2 and heatmap packages were used to create volcano maps and heatmaps of differentially expressed lncRNAs and mRNAs to make them visible.

### Co-expression network analysis of differentially expressed lncRNAs and mRNAs in patients with HCM

The Pearson correlation analysis of the differentially expressed lncRNAs and mRNAs was performed by the cor () function in the R language. The absolute value of the Pearson correlation coefficient ≥0.75 of lncRNA-mRNA pairs was considered to be significantly correlated and selected in the co-expression network. Subsequently, the co-expression network was visualized using Cytoscape software.

### Gene ontology (GO) enrichment and Kyoto Encyclopedia of genes and genomes (KEGG) pathway analysis of genes in the network

The functions of genes enriched in the co-expression network, including cellular components, biological processes, and molecular functions, were determined by using gene ontology (GO) enrichment analysis. The enriched pathways of genes were analyzed using the Kyoto Encyclopedia of Genes and Genomes (KEGG) pathway analysis. ClusterProfiler package in R was applied to GO enrichment and KEGG pathway analyses with a threshold of adjusted *p*-value < 0.05.

### Protein–protein interaction (PPI) network analysis of genes in the network

To predict the physical and functional interactions of proteins, protein–protein interaction (PPI) networks were constructed by the online Search Tool for the Retrieval of Interacting Genes/Proteins (STRING), and the combined score >0.4 was used as the cutoff criterion. Following the construction of the PPI network, the Cytohubba plugin in Cytoscape software was adopted to calculate the degree of each protein node. In this study, the top 10 nodes were regarded to be hub genes.

### Plasma collection and real-time quantitative PCR (qPCR)

The research design, protocol, and the use of human plasma samples were approved by the Medical Ethics Committee of the Xiangya Hospital of Central South University. The diagnosis of HCM was carried out according to the European Society of Cardiology Guidelines (21). 4 patients diagnosed with HCM and 4 healthy controls were included in this study. The clinical characteristics of the patients are listed in Supplementary Table 4. The peripheral whole blood of patients was collected in a tube containing an EDTA anticoagulant after overnight fasting, and plasma was separated by centrifugation. Total RNA was extracted from plasma samples using a miRNeasy Serum/Plasma Kit (Qiagen) according to the manufacturer's instructions. The quality and concentration of RNA were evaluated using NanoDrop 2000 spectrophotometer (Thermo Fisher Scientific). Complementary DNAs (cDNAs) were synthesized from the total RNA using the PrimeScript RT reagent Kit (Takara) in accordance with the manufacturer's instructions. The lncRNAs were quantified by performing real-time qPCR on a QuantStudio 5 Real-Time PCR System with SYBR Premix Ex Taq assays (Takara). The fold difference in the expression level between the two groups was calculated using the 2^−ΔΔCt^ method. The primer sequences are summarized in Supplementary Table 5.

### Statistical analysis

The results are expressed as mean±SD. The *T*-tests or nonparametric tests were used to compare the differences between the HCM group and the control group. Graphs were constructed with R software, GraphPad Prism, and online visualization tools. A two-tailed *P*-value of < 0.05 was considered to be statistically significant. Statistical analyses were done using GraphPad Prism 8.0 and SPSS 20.0.

## Results

### Identification of differentially expressed lncRNAs and mRNAs in patients with HCM compared with the controls

**Figure 1:**
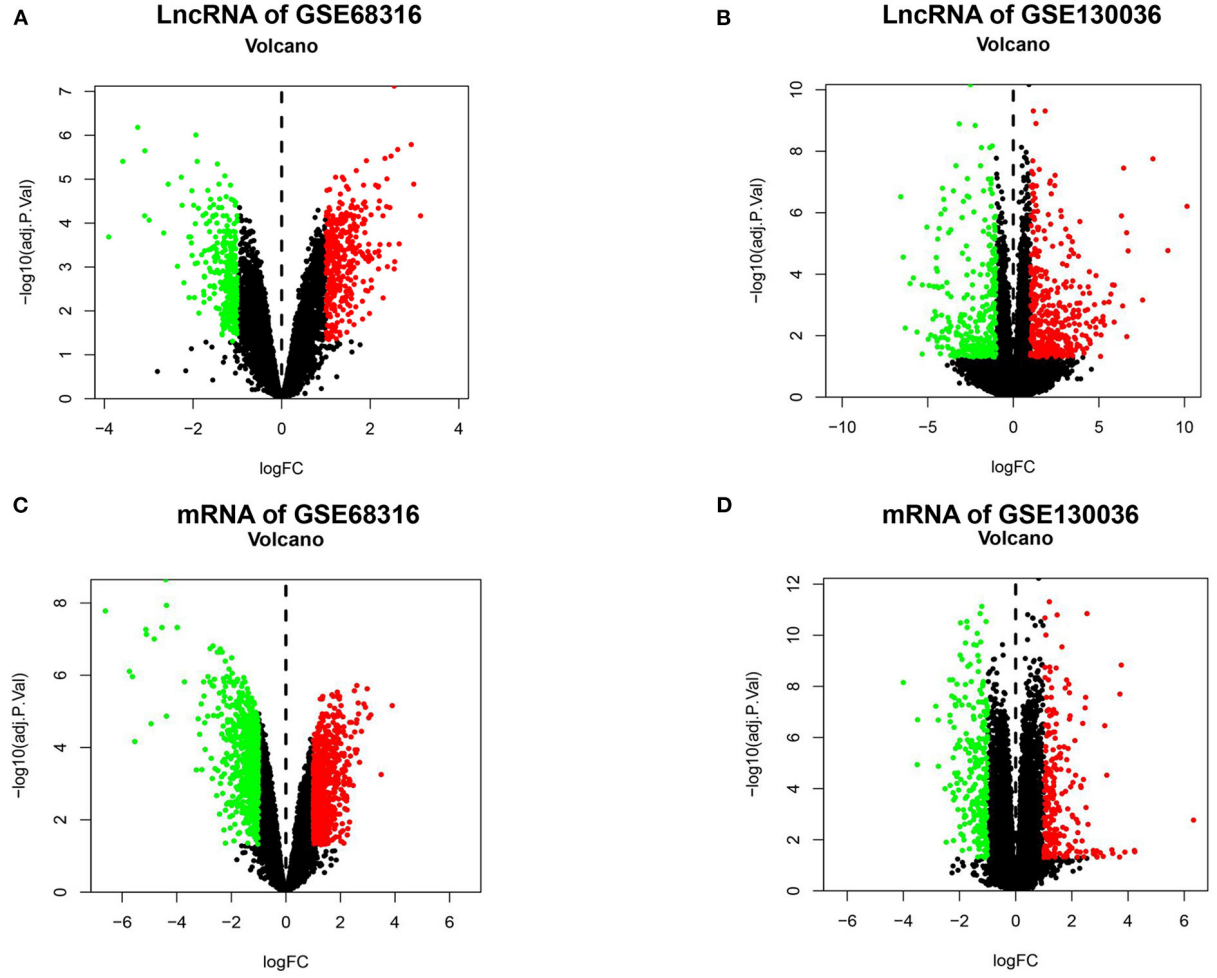
A Volcano plot of all the differentially expressed lncRNAs and mRNAs between patients with HCM and the controls. **(A)** A volcano plot of differentially expressed lncRNAs in the GSE68316 dataset. **(B)** A volcano plot of differentially expressed lncRNAs in the GSE130036 dataset. **(C)** A volcano plot of differentially expressed mRNAs in the GSE68316 dataset. **(D)** A volcano plot of differentially expressed mRNAs in GSE130036 dataset. Green dots represent differentially expressed genes with log-fold change <-1, red dots represent differentially expressed genes with log-fold change >1, and black spots represent genes with no significant difference in expression, adjusted *p*-value <0.05.

**Figure 2:**
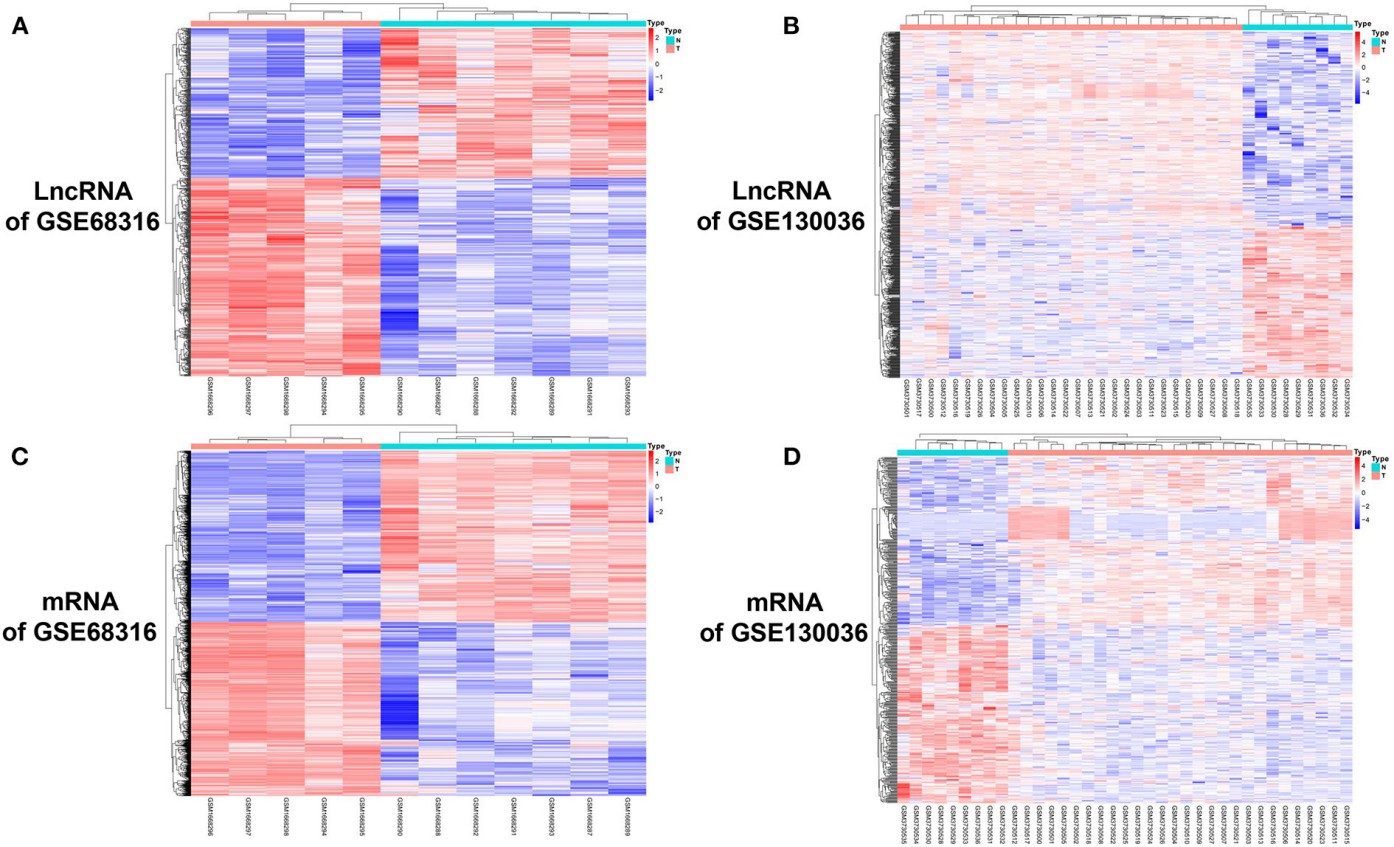
A cluster heat map of differentially expressed lncRNAs and mRNAs between HCM patients and the controls. **(A)** A heat map of differentially expressed lncRNAs in the GSE68316 dataset. **(B)** A heat map of differentially expressed lncRNAs in the GSE130036 dataset. **(C)** A heat map of differentially expressed mRNAs in the GSE68316 dataset. **(D)** A heat map of differentially expressed mRNAs in the GSE130036 dataset. The gradient color change from blue to red represents the changing process from downregulation to upregulation.

The gene expression data of HCM were obtained from GSE68316 and GSE130036 datasets, which included 35 patients with HCM and 14 controls, and the basic information of the datasets is presented in Supplementary Table 1. After normalization, a total of 1,426 differential expressed lncRNAs and 1,715 differential expressed mRNAs were obtained from GSE68316 (adjusted *P* < 0.05, fold change >2). Among them, 965 lncRNAs were upregulated and 461 lncRNAs were downregulated; 896 mRNAs were upregulated and 819 mRNAs were downregulated. The volcano plots of differentially expressed genes are shown in Figures 1A,C, and the cluster heatmaps are shown in Figures 2A,C. A total of 469 differentially expressed lncRNAs and 2,407 differentially expressed mRNAs were obtained from GSE130036, of which 183 lncRNAs and 1,283 mRNAs were upregulated and 286 lncRNAs and 1,124 mRNAs were downregulated (adjusted *P* < 0.05, fold change >2). The volcano plots of differentially expressed genes in GSE130036 are shown in Figures 1B,D, and the cluster heatmaps are shown in Figures 2B,D. The top 10 differentially expressed lncRNAs involved in the HCM of the two datasets are listed in Table 1, and the top 10 differentially expressed mRNAs are listed in Table 2.

**Table 1:** Top 10 upregulated and downregulated long noncoding RNAs in the myocardial tissues of patients with HCM in GSE68316 and GSE130036.

**GSE68316**					**GSE130036**				
**Ensembl_Gene_ID**	**Gene symbol**	**Adjust P**	**Regulation**	**Log2 FC**	**Ensembl Gene_ID**	**Gene symbol**	**Adjust P**	**Regulation**	**Log2 FC**
ENST00000445814	XIST	0.12802	up	3.89686	ENST00000624710	AC008522.1	0.01136	up	6.80673
ENST00000440196	LOC101928626	3.48E-21	up	3.69278	ENST00000611237	RP5-881P19.7	0.01272	up	6.62029
ENST00000588634	CTC-510F12.2	1.01E-15	up	3.32935	ENST00000619449	MALAT1-215	0.01494	up	4.98358
ENST00000472913	PLCH1-AS2	5.11E-19	up	3.05175	ENST00000489821	B3GALT5-AS1	0.02867	up	4.88654
ENST00000453100	LINC00570	8.53E-14	up	2.79108	ENST00000553348	CTD-2243E23.1	7.79E-06	up	4.73740
ENST00000549241	RP11-449P15.1	1.23E-15	up	2.74094	ENST00000507108	LINC02433	0.00069	up	4.67747
ENST00000558994	CTD-2308G16.1	1.37E-08	up	2.58255	ENST00000554055	RP11-841O20.2	0.01226	up	4.22569
ENST00000524768	CTD-2530H12.2	7.62E-13	up	2.52511	ENST00000425630	LINC00200	0.01358	up	4.16374
ENST00000456450	AC010907.2	2.23E-20	up	2.47399	ENST00000562361	AC018767.2	0.00044	up	3.90221
ENST00000420902	RP1-29C18.8	3.55E-10	up	2.47292	ENST00000554759	RP11-588P7.2	0.00076	up	3.78938
ENST00000450016	LINC01952	1.92E-07	down	2.81115	ENST00000634611	AC107068.2	0.00149	down	5.39759
ENST00000443565	LINC01781	0.00021	down	2.73194	ENST00000446593	AC093642.6	0.00292	down	5.27501
ENST00000376482	AC073842.19	4.56E-16	down	2.53420	ENST00000552470	RP11-632B21.1	9.28E-06	down	5.10923
ENST00000563833	AF213884.2	3.97E-09	down	2.33589	ENST00000430181	TSPEAR-AS1	4.24E-07	down	4.10253
ENST00000475250	PPP1R35-AS1	0.00086	down	2.29869	ENST00000555146	RP11-110A12.2	0.00019	down	3.97233
ENST00000424948	RP1-92O14.3	1.30E-06	down	2.21711	ENST00000453554	RP11-108M9.3	1.27E-14	down	3.88847
ENST00000570843	RP11-473M20.16	1.43E-16	down	2.20565	ENST00000330490	TSPEAR-AS2	4.76E-12	down	3.57308
ENST00000367477	STXBP5	0.00368	down	2.20057	ENST00000521558	RP11-1081M5.1	1.69E-24	down	3.49851
ENST00000507727	ALG14	4.59E-09	down	2.16974	ENST00000533578	FAM167A-AS1	0.03743	down	3.43473
ENST00000549023	AC010173.1	4.11E-08	down	2.14412	ENST00000467995	LINC00881	2.62E-16	down	3.42541

Log2 FC, Log2-fold change; adjust P, adjusted P-value; XIST, X Inactive Specific Transcript; PLCH1-AS2, PLCH1 Antisense RNA 2; PPP1R35-AS1, PPP1R35 Antisense RNA 1; STXBP5, Syntaxin Binding Protein 5; ALG14, ALG14 UDP-N-Acetylglucosaminyltransferase Subunit; MALAT1, metastasis-associated lung adenocarcinoma transcript 1; B3GALT5-AS1, B3GALT5 Antisense RNA 1; TSPEAR-AS1, TSPEAR Antisense RNA 1; TSPEAR-AS2, TSPEAR Antisense RNA 2; FAM167A-AS1, FAM167A Antisense RNA 1.

**Table 2:** Top 10 upregulated and downregulated mRNAs in the myocardial tissues of patients with HCM in GSE68316 and GSE130036.

**GSE68316**					**GSE130036**				
**Ensembl_Gene_ID**	**Gene symbol**	**Adjust P**	**Regulation**	**Log2 FC**	**Ensembl Gene_ID**	**Gene symbol**	**Adjust P**	**Regulation**	**Log2 FC**
ENST00000463664	SELENBP1	2.22E-17	up	6.481273	ENST00000368742	LORICRIN	2.26E-33	up	24.72548
ENST00000171887	TNS1	1.91E-13	up	6.36043	ENST00000437231	PCBP2	4.21E-34	up	24.54893
ENST00000330597	HBG1	3.36E-06	up	5.848873	ENST00000252244	KRT1	7.45E-28	up	23.59247
ENST00000380327	TROAP	6.80E-17	up	5.734056	ENST00000553458	ALDH6A1	6.28E-23	up	22.89827
ENST00000555156	LOC100506767	4.26E-17	up	5.246619	ENST00000396934	BTN3A2	3.58E-23	up	22.85187
ENST00000397027	EPB42	1.72E-18	up	5.11782	ENST00000425460	MYH14	3.93E-19	up	22.57398
ENST00000523022	CA1	1.24E-13	up	5.066804	ENST00000368295	ECHDC1	1.45E-16	up	22.04711
ENST00000399246	SLC4A1	8.42E-15	up	4.797823	ENST00000611477	ZNF16	6.11E-09	up	21.68905
ENST00000472539	HBM	2.08E-18	up	4.557216	ENST00000357992	ELK4	4.60E-08	up	21.62953
ENST00000537904	PDE4DIP	7.35E-19	up	4.386079	ENST00000585156	PDE4DIP	5.98E-08	up	21.46707
ENST00000463664	COMMD6	2.02E-19	down	3.84337	ENST00000504584	CORIN	1.42E-12	down	25.772
ENST00000309170	P2RY14	7.18E-13	down	3.299083	ENST00000509536	TECRL	1.54E-11	down	25.6542
ENST00000435402	CCDC7	4.90E-17	down	3.080423	ENST00000327705	BTNL9	1.10E-10	down	24.6503
ENST00000356719	KIAA1841	1.26E-19	down	3.010909	ENST00000360162	ADD3	3.04E-10	down	24.1061
ENST00000480956	LSM5	2.31E-17	down	2.997625	ENST00000613142	SELENOI	6.17E-10	down	23.7528
ENST00000293842	RPL26	6.68E-20	Down	2.935435	ENST00000503821	CORIN	0.00211	down	10.5294
ENST00000467106	RPS24	6.91E-19	down	2.902729	ENST00000370046	KCNIP2	0.000273	down	9.13034
ENST00000460380	C17orf108	1.51E-13	down	2.793557	ENST00000618099	FURIN	3.10E-06	down	8.64193
ENST00000496387	UQCRH	5.80E-17	down	2.764366	ENST00000392179	NDUFS2	0.002885	down	8.56594
ENST00000552548	PFDN5	2.17E-17	down	2.682237	ENST00000343195	KCNIP2	7.95E-05	down	8.36729

Log2 FC, Log2-fold change; adjust P, adjusted P-value; SELENBP1, Selenium Binding Protein 1; TNS1, Tensin 1; HBG1, Hemoglobin Subunit Gamma 1; TROAP, Trophinin Associated Protein; EPB42, Erythrocyte Membrane Protein Band 4.2; CA1, Carbonic Anhydrase 1; SLC4A1, Solute Carrier Family 4 Member 1; HBM, Hemoglobin Subunit Mu; PDE4DIP, Phosphodiesterase 4D Interacting Protein; COMMD6, COMM Domain Containing 6; P2RY14, Phosphodiesterase 4D Interacting Protein; CCDC7, Coiled-Coil Domain Containing 7; RPL26, Ribosomal Protein L26; RPS24, Ribosomal Protein S24; C17orf108, LYR Motif Containing 9, LYRM9; UQCRH, Ubiquinol-Cytochrome C Reductase Hinge Protein; PFDN5, Prefoldin Subunit 5; PCBP2, Poly(RC) Binding Protein 2; KRT1, Keratin 1; ALDH6A1, Aldehyde Dehydrogenase 6 Family Member A1; BTN3A2, Butyrophilin Subfamily 3 Member A2; MYH14, Myosin Heavy Chain 14; ECHDC1, Ethylmalonyl-CoA Decarboxylase 1; ZNF16, Zinc Finger Protein 16; ELK4, ETS Transcription Factor ELK4; CORIN, Corin, Serine Peptidase; TECRL, Trans-2,3-Enoyl-CoA Reductase Like; BTNL9, Butyrophilin Like 9; ADD3, Adducin 3; SELENOI, Selenoprotein I; KCNIP2, Potassium Voltage-Gated Channel Interacting Protein 2; FURIN, Furin, Paired Basic Amino Acid Cleaving Enzyme; NDUFS2, NADH: Ubiquinone Oxidoreductase Core Subunit S2.

**Figure 3:**
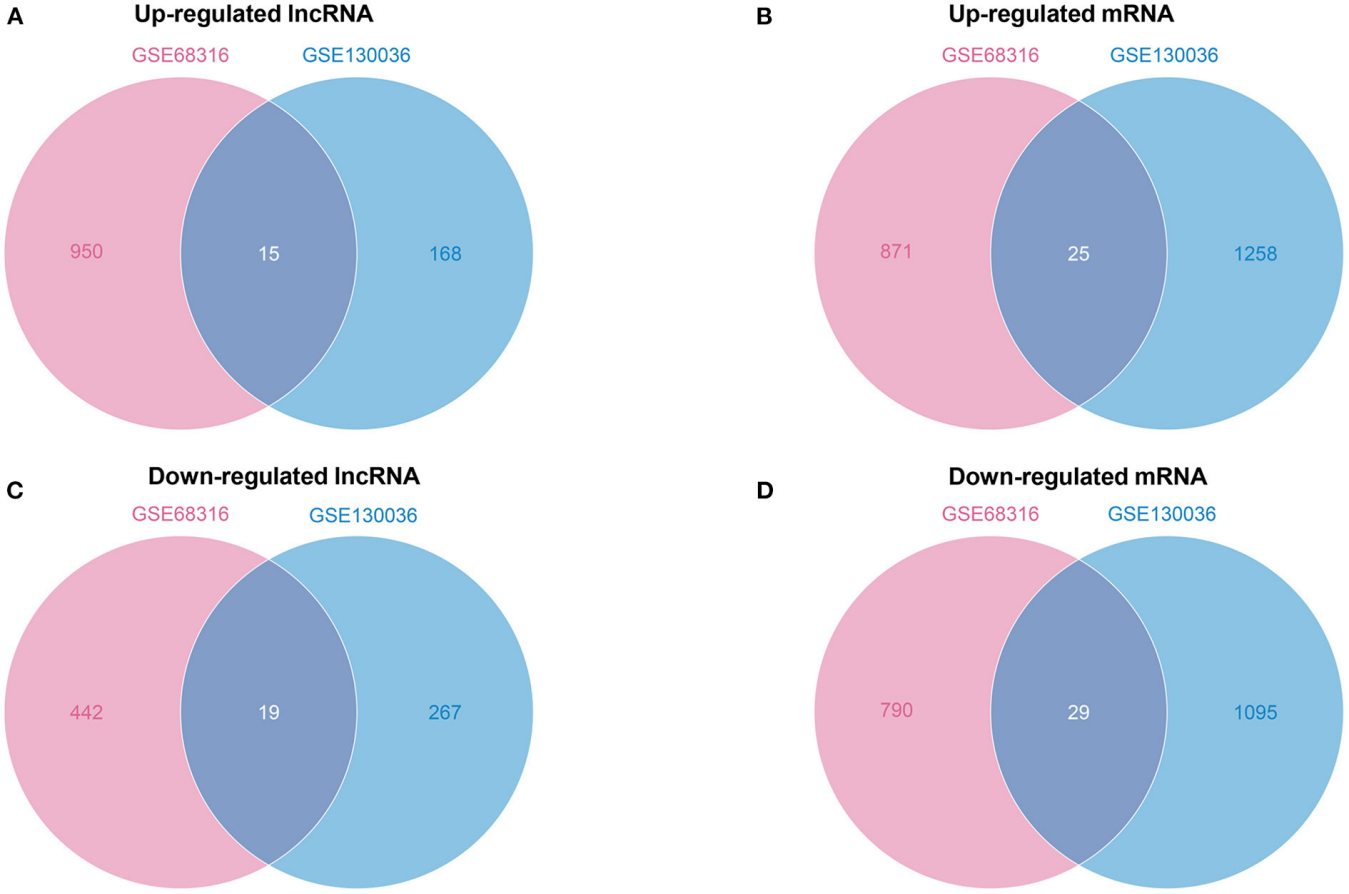
A Venn diagram of common differentially expressed lncRNAs and mRNAs. **(A)** A Venn diagram of upregulated common differentially expressed lncRNAs. **(B)** A Venn diagram of upregulated common differentially expressed mRNAs. **(C)** A Venn diagram of downregulated common differentially expressed lncRNAs. **(D)** A Venn diagram of downregulated common differentially expressed mRNAs.

Subsequently, the Venn diagrams of upregulated and downregulated genes are shown in Figure 3. A total of 34 common differentially expressed lncRNAs with 15 upregulated and 19 downregulated lncRNAs (Figures 3A,C) and a total of 54 common differentially expressed mRNAs with 25 upregulated and 29 downregulated mRNAs (Figures 3B,D) were thus identified.

### Differential lncRNA-mRNA co-expression network construction and analysis

**Figure 4:**
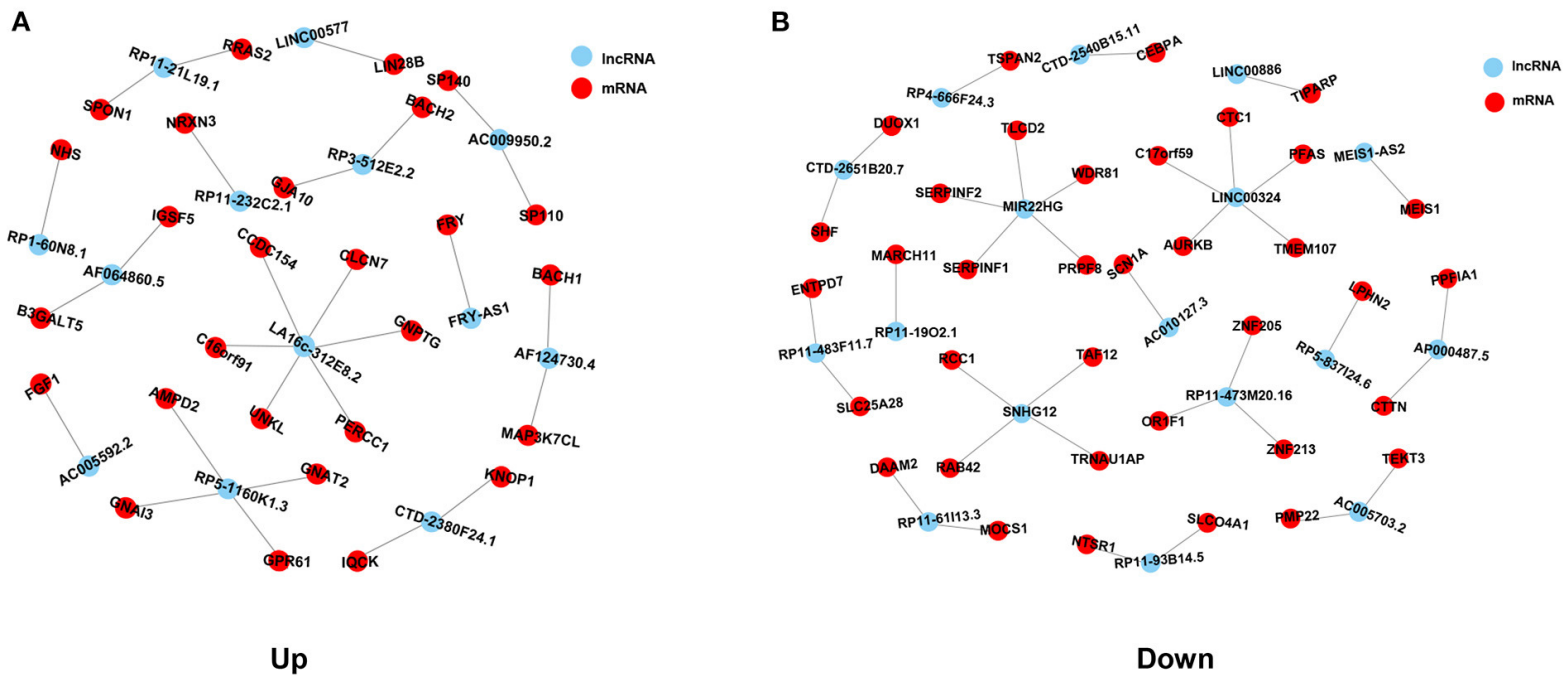
Co-expression network analysis of common differentially expressed lncRNAs and mRNAs in HCM. **(A)** Co-expression network of upregulated common differentially expressed lncRNAs and mRNAs. **(B)** Co-expression network of downregulated common differentially expressed lncRNAs and mRNAs. Red spots represent lncRNAs, blue spots represent mRNAs, and lines represent the co-expression relationships between lncRNAs and mRNAs.

To determine the function of differentially expressed lncRNAs and their role in HCM, a gene co-expression network between lncRNAs and mRNAs was constructed. Then, Pearson's correlation coefficients in all samples were calculated for correlation analysis of mRNAs and lncRNAs, and lncRNA-mRNA pairs with a coefficient of ≥0.75 were selected. A total of 63 lncRNA-mRNA pairs were observed to be involved in 30 differentially expressed lncRNAs (13 upregulated and 17 downregulated) and 63 differentially expressed mRNAs (27 in upregulated network and 36 in downregulated network). In the upregulated co-expression network, 27 lncRNA-mRNA pairs were observed with 13 differentially expressed lncRNAs and 27 mRNAs (Figure 4A). About 36 lncRNA-mRNA pairs were observed in the downregulated co-expression network, including 17 differentially expressed lncRNAs and 36 mRNAs (Figure 4B).

### GO function enrichment analysis of genes in the co-expression network

**Figure 5:**
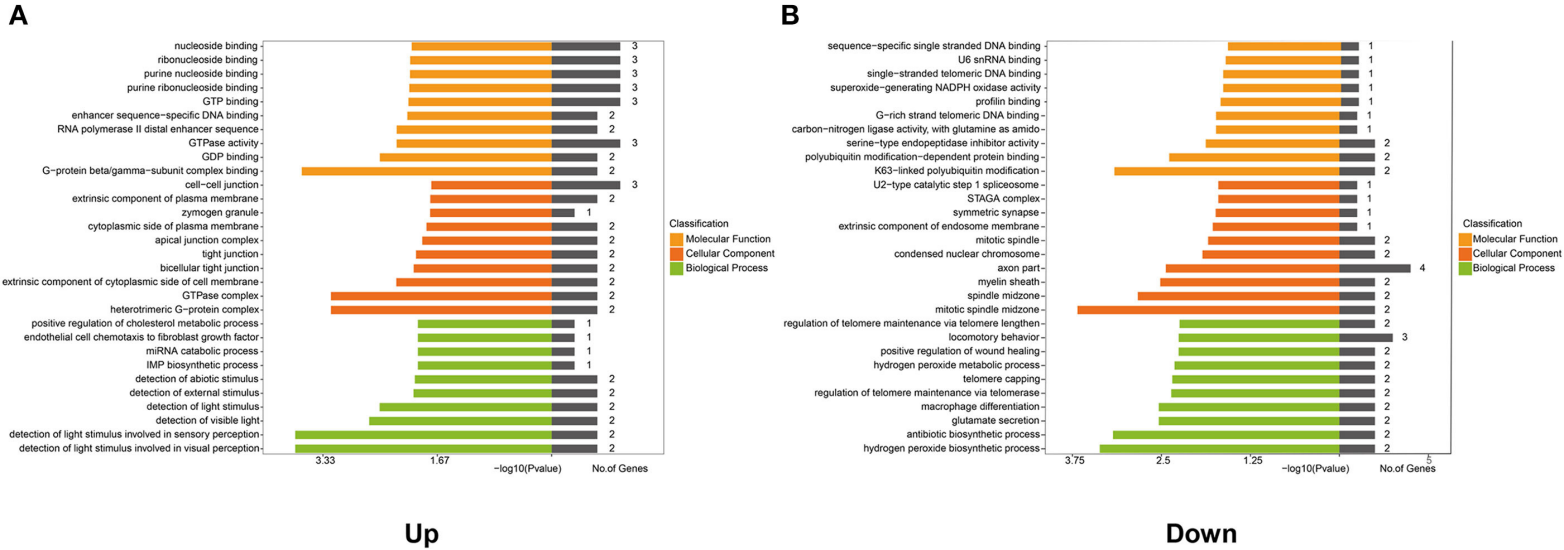
Gene Ontology (GO) analysis of genes in the co-expression network. GO analysis shows: **(A)** The top 10 enriched terms associated with the molecular function (MF), cellular component (CC), and biological process (BP) of genes in the upregulated co-expression network. **(B)** The top 10 enriched terms associated with MF, CC, and BP of genes in the downregulated co-expression network.

Furthermore, to ascertain the potential functions of the identified differentially expressed lncRNAs and mRNAs in the co-expression network, GO function enrichment analysis was performed (Figure 5). GO analysis mainly described three categories of results: ‘molecular functions' (MF), ‘cellular components' (CC), and ‘biological processes' (BP). The analyses exhibited the top 10 significant enrichment of MF, CC, and BP in terms of upregulated and downregulated genes in the co-expression network. As shown in Figure 5A, the enrichment analysis of upregulated co-expression network related to molecular functions was primarily enriched in G-protein beta/gamma-subunit complex binding (*P* = 0.0002), GDP binding (*P* = 0.0031), and GTPase activity (*P* = 0.0055), and the analysis related to cellular components were mainly enriched in heterotrimeric G-protein complex (*p* = 0.0006), GTPase complex (*P* = 0.0006), and the extrinsic component of the cytoplasmic side of the plasma membrane (*P* = 0.0005). Additionally, the analysis of biological processes showed that genes were mainly involved in the positive regulation of cholesterol metabolic process (*P* = 0.0112), endothelial cell chemotaxis to fibroblast growth factor (*P* = 0.0112), and miRNA catabolic process (*P* = 0.0112). On the other hand, the enrichment analysis related to the molecular functions of downregulated co-expression network focused on K63-linked polyubiquitin modification-dependent protein binding (*P* = 0.0007), polyubiquitin modification-dependent protein binding (*P* = 0.0041), and serine-type endopeptidase inhibitor activity (*P* = 0.0132). Also, the cellular components were primarily enriched in mitotic spindle midzone (*P* = 0.0002), spindle midzone (*P* = 0.0015), and myelin sheath (*P* = 0.0030). In regard to biological process enrichment analysis of downregulated co-expression network, genes were mainly involved in the hydrogen peroxide biosynthetic process (*P* = 0.0004), the antibiotic biosynthetic process (*P* = 0.0007), and glutamate secretion (*P* = 0.0029) (Figure 5B).

### KEGG pathway enrichment analysis of genes in the co-expression network

**Figure 6:**
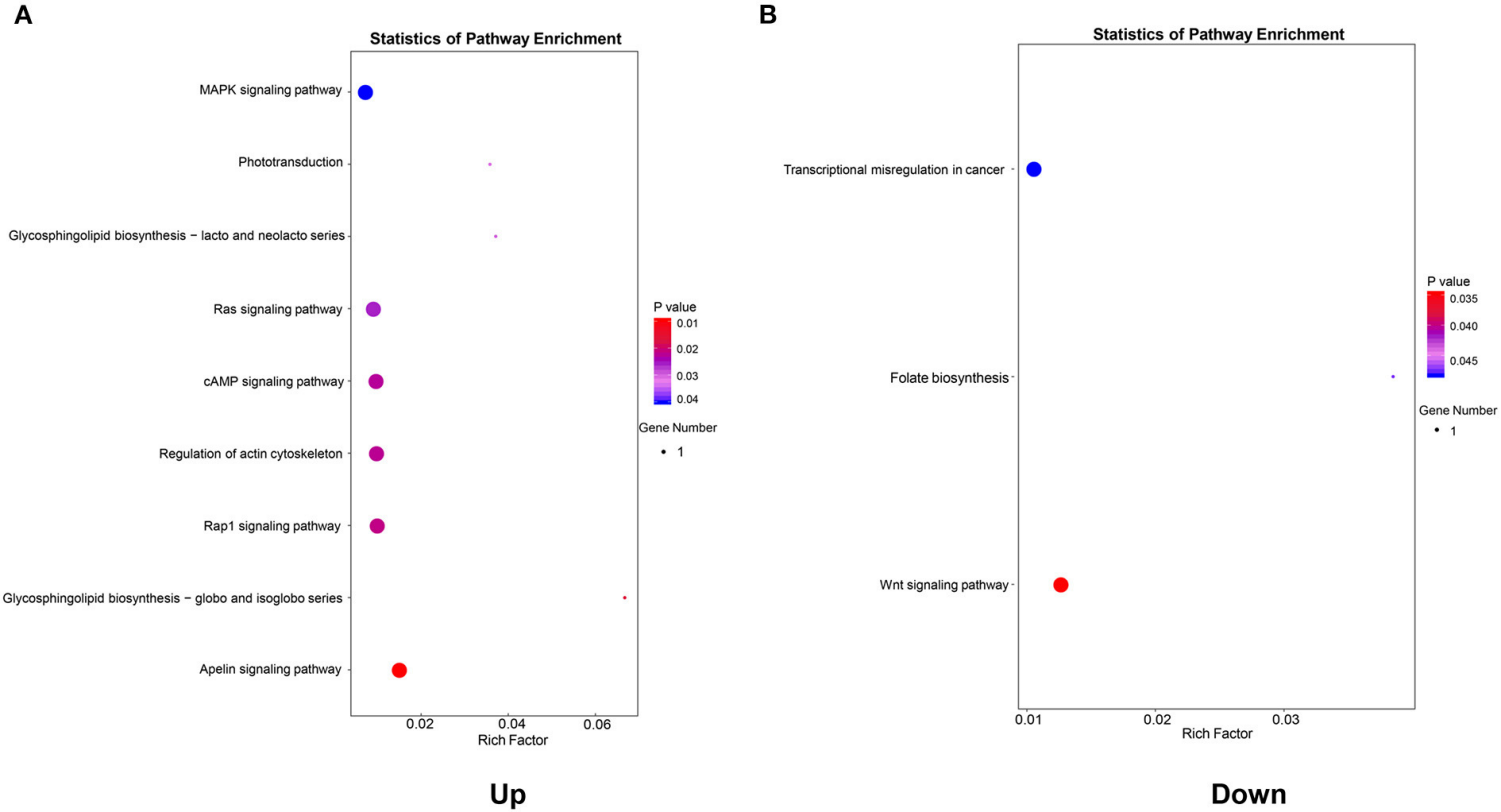
Kyoto Encyclopedia of Genes and Genomes (KEGG) pathway enrichment analysis of genes in the co-expression network. **(A)** KEGG pathways enriched by genes in the upregulated co-expression network. **(B)** KEGG pathways enriched by genes in the downregulated co-expression network.

To further identify the biological processes associated with genes in the co-expression networ, KEGG pathway enrichment analyses were performed. As shown in Figure 6A, a total of nine key pathways were found through the KEGG pathway enrichment analysis of upregulated genes, which were primarily enriched in the Apelin signaling pathway (*P* = 0.0095), the Rap1 signaling pathway (*P* = 0.0215), and the regulation of the actin cytoskeleton (*P* = 0.0223). However, the downregulated genes in the co-expression network were significantly enriched in the Wnt signaling pathway (*P* = 0.0347), the folate biosynthesis pathway (*P* = 0.0473), and the transcriptional misregulation pathway (*P* = 0.0483) (Figure 6B).

### PPI network construction analysis

**Figure 7:**
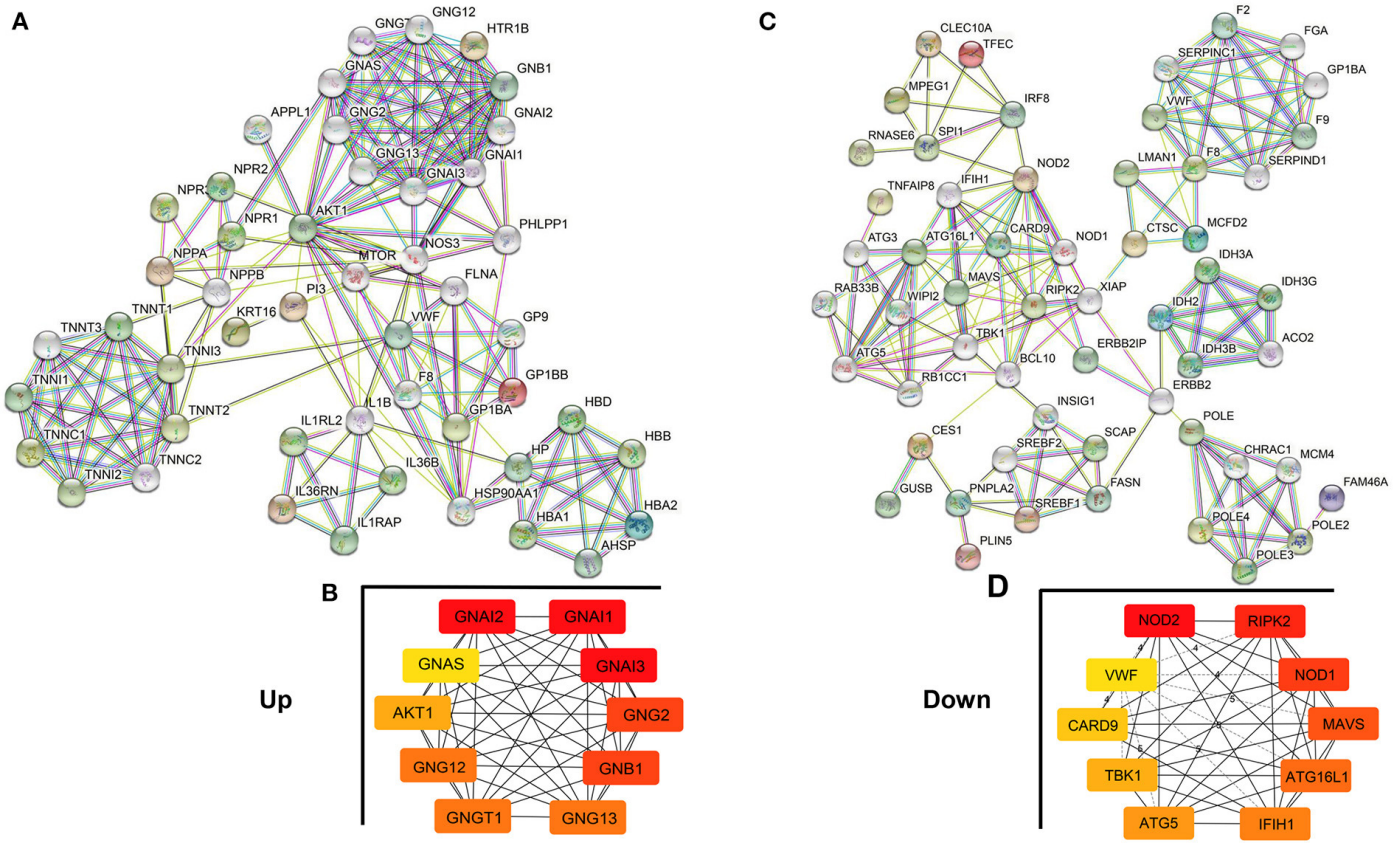
Protein–Protein Interaction (PPI) network analysis of genes in the co-expression network. **(A,B)** PPI network and hub genes in the upregulated co-expression network. **(C,D)** PPI network and hub genes in the downregulated co-expression network. The edges represent the interaction between two nodes, and the number of edges in one node is called the degree of the PPI network.

The PPI network analysis aims to study disease-related molecular mechanisms in co-expression networks and identify novel therapeutic targets from a systematic perspective. Consequently, a PPI network analysis of differentially expressed genes in the co-expression network was conducted with the STRING database. A total of 65 nodes and 362 edges were screened in the upregulated PPI network (Figure 7A). Nodes with high topological scores were considered to likely play an important role in disease, and G Protein Subunit Alpha I2 (GNAI2), G Protein Subunit Alpha I1 (GNAI1), G Protein Subunit Alpha I3 (GNAI3), G Protein Subunit Gamma 2 (GNG2), G Protein Subunit Beta 1 (GNB1), G Protein Subunit Gamma 13 (GNG13), G Protein Subunit Gamma Transducin 1 (GNGT1), G Protein Subunit Gamma 12 (GNG12), AKT Serine/Threonine Kinase 1 (AKT1), and GNAS Complex Locus (GNAS) were the top 10 hub genes in the PPI network of upregulated genes (Figure 7B, Supplementary Table 2). A total of 69 nodes and 314 edges were screened in the downregulated PPI network (Figure 7C). Nucleotide-Binding Oligomerization Domain Containing Protein 2 (NOD2), Receptor-Interacting Serine/Threonine Kinase 2 (RIPK2), Nucleotide-Binding Oligomerization Domain Containing 1 (NOD1), Mitochondrial Antiviral Signaling Protein (MAVS), Autophagy Related 16-Like 1 (ATG16L1), Interferon Induced With Helicase C Domain 1 (IFIH1), Autophagy Related 5 (ATG5), TANK Binding Kinase 1 (TBK1), Caspase Recruitment Domain Family Member 9 (CARD9), and von Willebrand factor (VWF) were the top 10 hub genes in the PPI network of downregulated genes (Figure 7D, Supplementary Table 2).

### Verification of crucial lncRNAs and mRNAs in patients with HCM

**Figure 8:**
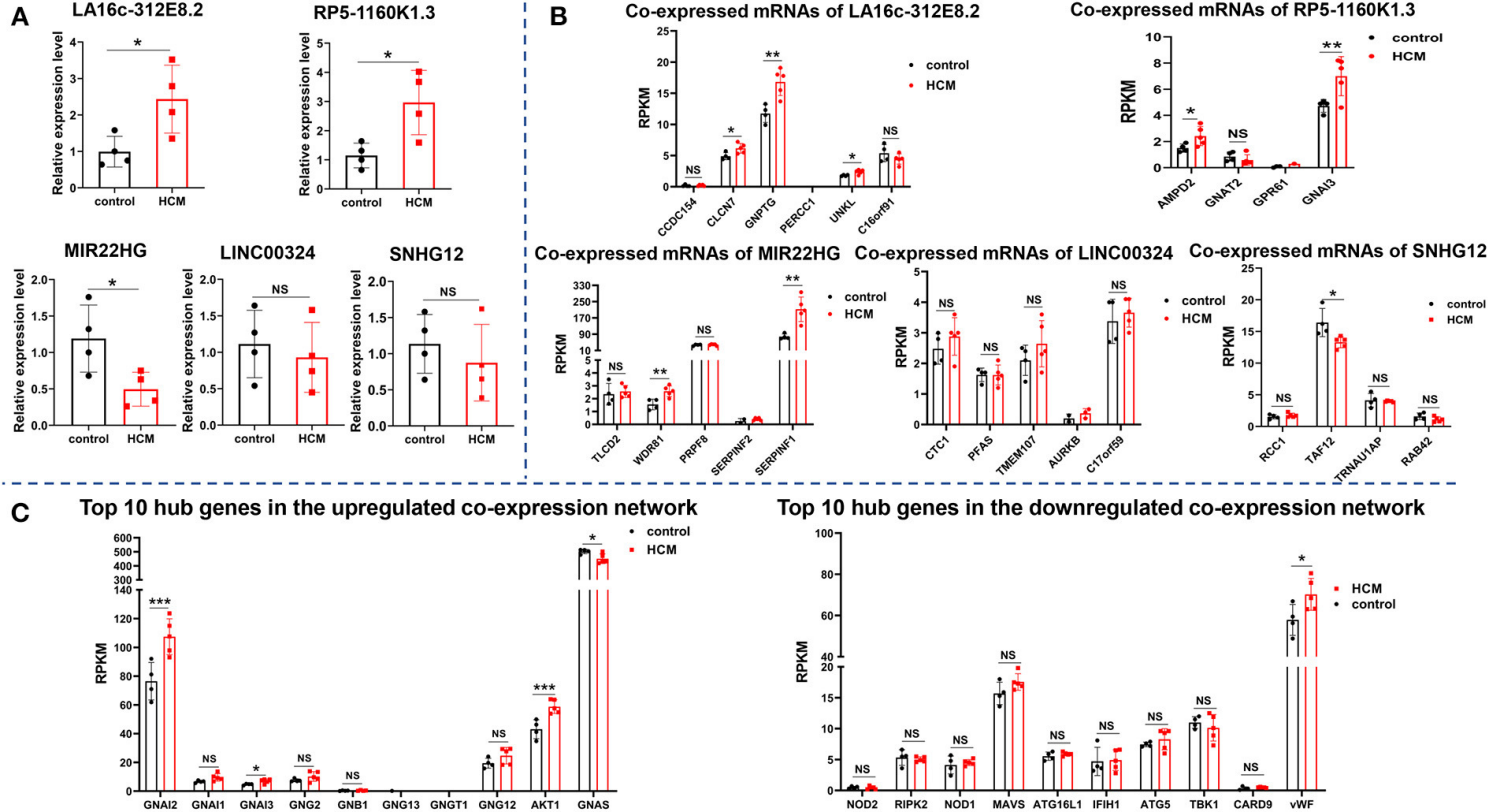
Verification of crucial lncRNAs and mRNAs in HCM patients. **(A)** The expression of five crucial lncRNAs in plasma of HCM patients and the controls were measured by real-time qPCR. **(B)** The expression level of mRNAs (co-expressed with five crucial lncRNAs) in the verification dataset GSE89714. **(C)** The expression level of the top 10 hub genes of upregulated and downregulated PPI network in the verification dataset GSE89714. ***p < 0.001.

In addition to expressing in specific tissues, lncRNA also exists stably in the circulating peripheral blood and hence can be used as biomarkers for the diagnosis and treatment of diseases (22). In order to verify the key lncRNAs and their diagnostic value for HCM, we collected plasma samples of patients with HCM and evaluated the level of five crucial lncRNAs (two upregulated and three downregulated) in the co-expression network by real-time qPCR. As shown in Figure 8A, lncRNA LA16c-312E8.2 (control: 0.99 ± 0.42 vs. HCM: 2.44 ± 0.93, *P* = 0.0303) and RP5-1160K1.3 (control: 1.15 ± 0.42 vs HCM: 2.97 ± 1.10, *P* = 0.0219) were upregulated and lncRNA MIR22 host gene (MIR22HG) (control: 1.19 ± 0.46 vs. HCM: 0.49 ± 0.23, *P* = 0.0360) was downregulated in patients with HCM, which was consistent with our previous analysis results. However, the levels of LINC00324 (control: 1.11 ± 0.46 vs. HCM: 0.93 ± 0.48, *P* = 0.6037) and Small Nucleolar RNA Host Gene 12 (SNHG12) (control: 1.14 ± 0.40 vs. HCM: 0.88 ± 0.53, *P* = 0.4657) were not significantly different between the two groups.

In addition to verify the expression of lncRNAs in peripheral blood, we selected GSE89714 as the validation dataset to verify the expression levels of mRNAs (co-expressed with five crucial lncRNAs) in the co-expression network and the top 10 hub genes in the PPI network between HCM and healthy control cardiac tissues. As seen in Figure 8B, Chloride Voltage-Gated Channel 7 (CLCN7), N-Acetylglucosamine-1-Phosphate Transferase Subunit Gamma (GNPTG), and Unk Like Zinc Finger (UNKL), which co-expressed with LA16c-312E8.2, were upregulated in the cardiac tissue of patients with HCM, whereas Coiled-Coil Domain Containing 154 (CCDC154), Proline and Glutamate Rich with Coiled Coil 1 (PERCC1), and C16orf91 showed no significant differences between the two groups; LncRNA RP5-1160K1.3 co-expressed mRNAs Adenosine Monophosphate Deaminase 2 (AMPD2) and GNAI3 were upregulated, and G Protein Subunit Alpha Transducin 2 (GNAT2) and G Protein-Coupled Receptor 61 (GPR61) showed no significant changes. In the co-expressed mRNAs of MIR22HG, WD Repeat Domain 81 (WDR81) and Serpin Family F Member 1 (SERPINF1) were upregulated in the HCM group, and TLC Domain Containing 2 (TLCD2), Pre-mRNA Processing Factor 8 (PRPF8), and Serpin Family F Member 2 (SERPINF2) showed no significant differences. In the co-expressed mRNAs of SNHG12, TATA-Box Binding Protein Associated Factor 12 (TAF12) was downregulated in HCM cardiac tissues, and other genes like Regulator of Chromosome Condensation 1 (RCC1), TRNA Selenocysteine 1 Associated Protein 1 (TRNAU1AP), and RAB42 showed no significant differences. Unfortunately, there were no significant differences in the expression of LINC00324 co-expressed mRNAs, which include CST telomere replication complex component 1 (CTC1), Phosphoribosylformylglycinamidine synthase (PFAS), Transmembrane Protein 107 (TMEM107), Aurora Kinase B (AURKB), and C17orf59, compared with the control group. In regard to the top 10 hub genes, the analysis of the dataset GSE89714 found that GNAI2, GNAI3, and AKT1 were upregulated, whereas GNAS was downregulated in the upregulated PPI network. Other genes, including GNAI1, GNG2, GNB1, GNG13, GNGT1, and GNG12, showed no significant differences between HCM and control cardiac tissue. In the downregulated network, only one gene (vWF) was statistically different (Figure 8C).

## Discussion

Hypertrophic cardiomyopathy, a type of hereditary cardiomyopathy, is the most common risk factor for sudden death in young people. With the emergence of next-generation sequencing (NGS) technologies, the list of variants and genes implicated in HCM is also expanding. These discoveries allow the precise identification of at-risk individuals prior to clinical diagnosis and provide novel therapeutic approaches for the modulation and prevention of HCM (23–26). A growing number of studies showed that lncRNAs play a momentous regulatory function in the pathophysiology of cardiovascular diseases, such as acute myocardial infarction, heart failure, and atrial fibrillation (15–17). Sequencing studies gradually found that lncRNAs also play an important role in the pathogenesis of HCM (27, 28), but the comprehensive evaluation of multiple datasets has not been thoroughly investigated. In the present study, we identified a total of 1,861 lncRNAs (1,133 upregulated and 728 downregulated) and 4,068 mRNAs (2,154 upregulated and 1,914 downregulated) were differentially expressed between the HCM and controls in the GSE68316 and GSE130036 datasets. Among these differentially expressed genes, 34 lncRNAs (15 upregulated and 19 downregulated) and 54 mRNAs (25 upregulated and 29 downregulated) were the commonly found differentially expressed genes. Co-expression network construction and subsequent GO enrichment and KEGG pathway analysis showed that the upregulated co-expression network was mainly enriched in G-protein beta/gamma-subunit complex binding, heterotrimeric G-protein complex, the Apelin signaling pathway, and the Rap1 signaling pathway. In addition, the downregulated network was mainly enriched in K63-linked polyubiquitin modification-dependent protein binding, mitotic spindle midzon, the Wnt signaling pathway, and the folate biosynthesis pathway. Of note, plasma sample validation of patients with HCM prompts that three key lncRNAs (LA16c-312E8.2, RP5-1160K1.3, and MIR22HG) may serve as biomarkers and intervention targets in HCM. These analyses provide novel insights to explore the potential mechanisms underlying HCM progression.

LncRNA has been found to regulate cardiomyocyte hypertrophy, apoptosis, angiogenesis, atherosclerosis, and other pathophysiological processes and play an important role in the development of cardiovascular disease (29–32). In recent years, it has also been reported that lncRNA plays an important role in HCM. Janika Viereck et al. reported that LncRNA H19 is highly conserved and downregulated in the failing hearts of mice, pigs, and humans. The H19 gene therapy prevents and reverses experimental pressure-overload-induced heart failure according to interaction with the polycomb repressive complex 2, suppressing H3K27 trimethylation, which, in turn, leads to reduced NFAT expression and activity (33). On the other hand, Xiao et al. (34) found that lncRNA X Inactive Specific Transcript (XIST) expression was significantly upregulated in hypertrophic mouse hearts and phenylephrine-treated cardiomyocytes. XIST promoted the progression of cardiac hypertrophy through competitively binding with miR-101 to enhance the expression of TLR2. After knocking down XIST, PE-induced cardiomyocyte hypertrophy was attenuated. In our research, a total of 1861 differentially expressed lncRNAs were identified by integrating datasets GSE68316 and GSE130036, among which 34 lncRNAs were expressed in both datasets. These common differentially expressed lncRNAs were analyzed to be co-expressed with several genes.

LncRNA LA16c-312E8.2 is also named LOC101929440. In addition to upregulated expression in HCM, LA16c-312E8.2 has also been reported to be differentially expressed in HER-2 enriched subtypes of breast cancer and pancreatic cancer (35, 36). However, the exact function and regulatory role of LA16c-312E8.2 has not yet been studied. The mRNAs co-expressed with LA16C-312E8.2 included CCDC154, CLCN7, GNPTG, PERCC1, UNKL, and C16orf91. CCDC154 is mainly involved in osteopetrosis and hypoplastic left heart syndrome (37, 38), and CLCN7 is mainly involved in osteopetrosis and angiogenesis (39). Diseases associated with GNPTG include mucolipidosis III gamma and mucolipidosis (40, 41), and those associated with PERCC1 include diarrhea 11, malabsorptive, congenital, and hepatocellular carcinoma (42–44). UNKL is associated with mucolipidosis (45), while the function and role of C16orf91 have not been reported. Among these co-expressed mRNAs, GNPTG and UNKL were involved in mucolipidosis that is associated with dilated cardiomyopathy in mucolipidosis type 2 (46), and CLCN7 was related to angiogenesis (39). These three genes are associated with cardiovascular diseases but have not been reported in HCM, which may be related to the lack of adequate research on HCM. In this study, our verification results showed that LncRNA LA16c-312E8.2 was upregulated in the plasma samples of patients with HCM compared to the controls. Besides, verification analysis of the public dataset showed that CCDC154, GNPTG, and UNKL were upregulated in the heart tissues of patients with HCM in the GSE89714 dataset, but there were no significant differences in other genes between the patients with HCM and controls.

MIR22HG, also known as C17orf91, is a downregulated lncRNA in HCM. The actions and functions of the MIR22HG gene are complex and have not been fully elucidated, but it has been reported to be involved in the regulation of cell proliferation and death according to several signaling pathways, including Wnt/β-catenin, epithelial-mesenchymal transition (EMT), notch, and STAT3 (47, 48). These pathways may promote the process of myocardial fibrosis in HCM. Previous studies reported that MIR22HG aggravates hypoxia-induced injury in cardiomyocytes and endothelial cells (49, 50); however, the exact mechanism of this gene in HCM has not been elucidated. MIR22HG was co-expressed with SERPINF1, SERPINF2, TLCD2, WDR81, and PRPF8. SERPINF1 and SERPINF2 are members of the serpin family. SERPINF1 strongly inhibits angiogenesis, and SERPINF2 is involved in alpha-2-plasmin inhibitor deficiency, vasculitis, and left ventricular diastolic dysfunction (51, 52). TLCD2 participates in Chromosome 17P13.3, Centromeric, Duplication Syndrome and is reported to be associated with increased left ventricular mass and cardiac hypertrophy (53). WDR81 is necessary for Purkinje and photoreceptor cell survival and has been reported to be associated with cerebellar ataxia and mental retardation. PRPF8 is essential for oxidative stress injury-induced mitophagy, which in turn leads to intracellular energy metabolism disorders (54). Although these genes have not been reported in HCM, they are involved in pathophysiological processes, including left ventricular diastolic dysfunction, myocardial hypertrophy, and disturbances in cellular energy metabolism, which are closely related to HCM. Therefore, it deserves our attention and further study.

We further analyzed the co-expression network of lncRNA-mRNA and found two hub gene networks. In the upregulated network, 10 hub genes were enumerated, which were involved in regulating the G protein-coupled receptor (GPCR) signaling pathway and GTPase activity. GPCR mediates many pathophysiological processes, such as cardiac contractility, hypertrophy, proliferation, survival, and fibrosis, which are closely correlated with HCM (55–57). Xin Liu et al. reported that ERK1/2 communicates with the GPCR signaling pathways to promote HCM upon Ang-II stimulation (58). In addition, Feng Xie et al. found that the GPCR family member APJ interacts with its ligand and promotes cardiac hypertrophy through the PI3K-autophagy and endoplasmic reticulum stress-autophagy pathways (59). Although the position of GCRP in cardiac hypertrophy is now well-recognized, the roles and mechanisms of GCRP family members in HCM remain poorly understood. Therefore, we need to focus on and study these genes and their related GPCR pathways in the next step. With regard to the downregulated network, 10 hub genes were filtered out, which were enriched in positive regulation of tumor necrosis factor production and nucleotide-binding oligomerization domain (NOD)-like receptor signaling pathway. A clinical study found that, after non-surgical septal reduction therapy for patients with hypertrophic obstructive cardiomyopathy, the expression of tumor necrosis factor-α was decreased and cardiac hypertrophy was regressed (60). Knockout of tumor necrosis factor-related protein reduced myocardial hypertrophy in mice (61). NOD leucine-rich repeat (LRR) protein family plays an important role in regulating the intracellular recognition of bacterial components by immune cells and is crucial for the maintenance of immune homeostasis (62). Jing Zong et al. found that NOD2 expression was upregulated in cardiomyocytes of aortic banding hypertrophic mice. NOD2 deficiency promoted cardiac hypertrophy and fibrosis by activating TLR4 and the MAPKs, NF-kB, and TGF-β/Smad pathways (63). Furthermore, the data showed that NOD-like receptor NLRP3 expression was downregulated in the aortic banding hypertrophic mice. NLRP3 deficiency accelerated cardiac hypertrophy, fibrosis, and inflammation responses with deteriorating cardiac dysfunction (64). Although the role of NOD in HCM is still controversial at present, its status and importance for cardiac hypertrophy have been widely recognized. Therefore, it is crucial to explore and elucidate the mechanism of these hub genes in HCM.

This study has certain limitations. Although both datasets contain several hundred differentially expressed lncRNAs and mRNAs, only a few of them overlapped. It is likely that the dominant mutant genotypes of the patients with HCM in the GSE130036 dataset were MYH7 and MYBPC3 mutations, while the dominant mutant genotypes of the patients with HCM in the GSE68316 dataset were not clear. It is well-known that numerous mutant genotypes lead to HCM, but pathophysiological processes caused by each genotype are not the same, resulting in different gene expressions in patients with different genotypes (65, 66). Notably, inconsistent sample size, sequencing methods, and baseline data of patients between the two datasets may also account for a few of the co-differentially expressed genes. In addition, due to the difficulties in obtaining cardiac tissues from patients with HCM, we did not validate the results in cardiac tissues. Finally, we could obtain limited plasma samples from patients with HCM for validation, and this may be the reason for the inconsistent results of lncRNA LINC00324 and SNHG12 between GEO analysis and our real-time qPCR data. In the future, more samples are needed to verify the abovementioned results.

## Conclusion

The present study aimed to analyze and elucidate the differentially expressed lncRNAs and mRNAs by integrative biological analysis of lncRNA-mRNA co-expression networks in patients with HCM. Three key lncRNAs (LA16c-312E8.2, RP5-1160K1.3, and MIR22HG) identified in patients with HCM may serve as biomarkers and intervention targets for HCM. These findings will provide a new thought in understanding the cause and mechanism of HCM and discovering new biomarkers or therapeutic targets for clinic treatment.

## Data availability statement

The datasets presented in this study can be found in online repositories. The names of the repository/repositories and accession number(s) can be found in the article/Supplementary material.

## Ethics statement

This study was reviewed and approved by the Committee at Xiangya Hospital of Central South University (IRB No. 202113548).

## Author contributions

JC conducted the statistical analysis, carried out the experiments, and drafted the article. LY contributed to reviewing, editing, and revising the article. All authors contributed to manuscript revision, read, and approved the submitted version.

## Funding

The work was supported by grants from Xiangya Hospital, Wei Ming Clinical, and Rehabilitation Research Fund of Peking University (No. xywmII09).

## Conflict of interest

The authors declare that the research was conducted in the absence of any commercial or financial relationships that could be construed as a potential conflict of interest.

## Publisher's note

All claims expressed in this article are solely those of the authors and do not necessarily represent those of their affiliated organizations, or those of the publisher, the editors and the reviewers. Any product that may be evaluated in this article, or claim that may be made by its manufacturer, is not guaranteed or endorsed by the publisher.

## Glossary

ADD3, Adducin 3; ALDH6A1, Aldehyde Dehydrogenase 6 Family Member A1; ALG14, ALG14 UDP-N-Acetylglucosaminyltransferase Subunit; AMPD2, Adenosine Monophosphate Deaminase 2; ANP, Atrial natriuretic peptide; ATG5, Autophagy Related 5; ATG16L1, Autophagy Related 16-Like 1; AURKB, Aurora Kinase B; B3GALT5-AS1, B3GALT5 Antisense RNA 1; BNP, Brain Natriuretic Peptide; BP, Biological processes; BTN3A2, Butyrophilin Subfamily 3 Member A2; BTNL9, Butyrophilin Like 9; C16orf91, Chromosome 16 Open Reading Frame 91; C17orf108, LYR Motif Containing 9, LYRM9; CA1, Carbonic Anhydrase 1; CARD9, Caspase Recruitment Domain Family Member 9; CCDC7, Coiled-Coil Domain Containing 7; CCDC154, Coiled-Coil Domain Containing 154; CLCN7, Chloride Voltage-Gated Channel 7; COMMD6, COMM Domain Containing 6; CORIN, Corin, Serine Peptidase; CTC1, CST telomere replication complex component 1; ECHDC1, Ethylmalonyl-CoA Decarboxylase 1; ELK4, ETS Transcription Factor ELK4; ENCODE, Encyclopedia of DNA Elements; EPB42, Erythrocyte Membrane Protein Band 4.2; FAM167A-AS1, FAM167A Antisense RNA 1; FURIN, Furin, Paired Basic Amino Acid Cleaving Enzyme; GEO, Gene Expression Omnibus; GO, Gene Ontology; GNAI1, G Protein Subunit Alpha I1; GNAI2, G Protein Subunit Alpha I2; GNAI3, G Protein Subunit Alpha I3; GNAT2, G Protein Subunit Alpha Transducin 2; GNB1, G Protein Subunit Beta 1; GNG2, G Protein Subunit Gamma 2; GNG12, G Protein Subunit Gamma 12; GNG13, G Protein Subunit Gamma 13; GNGT1, G Protein Subunit Gamma Transducin 1; GNAS, GNAS Complex Locus; GNPTG, N-Acetylglucosamine-1-Phosphate Transferase Subunit Gamma; HCM, Hypertrophic cardiomyopathy; HBG1, Hemoglobin Subunit Gamma 1; HBM, Hemoglobin Subunit Mu; IFIH1, Interferon Induced With Helicase C Domain 1; KEGG, Kyoto Encyclopedia of Genes and Genomes; KRT1, Keratin 1; KCNIP2, Potassium Voltage-Gated Channel Interacting Protein 2; LncRNAs, Long noncoding RNAs; LRR, leucine rich repeat; MF, molecular function; MAVS, Mitochondrial antiviral-signaling protein; MIR22HG, MIR22 Host Gene; MYBPC3, Myosin Binding Protein C3; MYH7, Myosin Heavy Chain 7; MYH14, Myosin Heavy Chain 14; ncRNA, noncoding RNA; NOD, Nucleotide-Binding Oligomerization Domain; NOD1, Nucleotide-Binding Oligomerization Domain Containing Protein 1; NOD2, Nucleotide-Binding Oligomerization Domain Containing 2; NDUFS2, NADH: Ubiquinone Oxidoreductase Core Subunit S2; P2RY14, Phosphodiesterase 4D Interacting Protein; PDE4DIP, Phosphodiesterase 4D Interacting Protein; PERCC1, Proline and Glutamate Rich with Coiled Coil 1; PFAS, Phosphoribosylformylglycinamidine synthase; PFDN5, Prefoldin Subunit 5; PLCH1-AS2, PLCH1 Antisense RNA 2; PPI, Protein–protein interaction; PPP1R35-AS1, PPP1R35 Antisense RNA 1; PCBP2, Poly(RC) Binding Protein 2; PRPF8, pre-mRNA processing factor 8; RCC1, Regulator of Chromosome Condensation 1; RIPK2, Receptor-Interacting Serine/Threonine Kinase 2; RPL26, Ribosomal Protein L26; RPS24, Ribosomal Protein S24; SELENBP1, Selenium Binding Protein 1; SERPINF1, Serpin Family F Member 2; SERPINF2, Serpin Family F Member 2; SLC4A1, Solute Carrier Family 4 Member 1; SELENOI, Selenoprotein I; STXBP5, Syntaxin Binding Protein 5; TAF12, TATA-Box Binding Protein Associated Factor 12; TBK1, TANK Binding Kinase 1; TECRL, Trans-2,3-Enoyl-CoA Reductase Like; TLCD2, TLC Domain Containing 2; TMEM107, Transmembrane Protein 107; TRNAU1AP, TRNA Selenocysteine 1 Associated Protein 1; TSPEAR-AS1, TSPEAR Antisense RNA 1; TSPEAR-AS2, TSPEAR Antisense RNA 2; TNS1, Tensin 1; TROAP, Trophinin Associated Protein; UNKL, Unk Like Zinc Finger; UQCRH, Ubiquinol-Cytochrome C Reductase Hinge Protein; VWF, von Willebrand factor; WDR81, WD Repeat Domain 81; XIST, X Inactive Specific Transcript; ZNF16, Zinc Finger Protein 16.
