# Supplementary material for: Identification of key genes for hypertrophic cardiomyopathy using integrated network analysis of differential lncRNA and gene expression
Source: Front Cardiovasc Med. 2022 Aug 4;9:946229. doi: 10.3389/fcvm.2022.946229 (PMC9386162; doi:10.3389/fcvm.2022.946229)
Supplement: Supplementary file 1 [file Table_1.docx]

**Supplementary table 1: Characteristics of datasets in HCM patients.**

| **Reference** | **Type** | **Sample** | **GEO** | **Platform** | **HCM** | **Control** |
| --- | --- | --- | --- | --- | --- | --- |
| Wei Yang, et al.  (2015) | LncRNA  mRNA | Human myocardial tissue of left ventricular septum | GSE68316 | GPL20113 | 7 | 5 |
| Xuanyu Liu, et al. (2019) | LncRNA  mRNA | Human myocardial tissue of left ventricular septum | GSE130036 | GPL20795 | 28 | 9 |
| Yulin Li, et al.  (2019) | mRNA | Human myocardial tissue of left ventricular septum | GSE89714 | GPL11154 | 5 | 4 |

Abbreviation: GEO, Gene Expression Omnibus; HCM, Hypertrophic cardiomyopathy.
