# Supplementary material for: Identification of key genes for hypertrophic cardiomyopathy using integrated network analysis of differential lncRNA and gene expression
Source: Front Cardiovasc Med. 2022 Aug 4;9:946229. doi: 10.3389/fcvm.2022.946229 (PMC9386162; doi:10.3389/fcvm.2022.946229)
Supplement: Supplementary file 2 [file Table_2.docx]

**Supplementary table 2: The top 10 hub genes in the protein-protein interaction (PPI) network of upregulated co-expressed mRNAs.**

| **Gene** | **Description** | **Node** |
| --- | --- | --- |
| GNAI2 | G Protein Subunit Alpha I2 | 12 |
| GNAI1 | G Protein Subunit Alpha I1 | 12 |
| GNAI3 | G Protein Subunit Alpha I3 | 12 |
| GNG2 | G Protein Subunit Gamma 2 | 11 |
| GNB1 | G Protein Subunit Beta 1 | 11 |
| GNG13 | G Protein Subunit Gamma 13 | 10 |
| GNGT1 | G Protein Subunit Gamma Transducin 1 | 10 |
| GNG12 | G Protein Subunit Gamma 12 | 10 |
| AKT1 | AKT Serine/Threonine Kinase 1 | 21 |
| GNAS | GNAS Complex Locus | 10 |
