# Supplementary material for: Identification of key genes for hypertrophic cardiomyopathy using integrated network analysis of differential lncRNA and gene expression
Source: Front Cardiovasc Med. 2022 Aug 4;9:946229. doi: 10.3389/fcvm.2022.946229 (PMC9386162; doi:10.3389/fcvm.2022.946229)
Supplement: Supplementary file 3 [file Table_3.docx]

**Supplementary table 3: The top 10 hub genes in the protein-protein interaction (PPI) network of downregulated co-expressed mRNAs.**

| **Gene** | **Description** | **Node** |
| --- | --- | --- |
| NOD2 | Nucleotide Binding Oligomerization Domain Containing 2 | 13 |
| RIPK2 | Receptor Interacting Serine/Threonine Kinase 2 | 11 |
| NOD1 | Nucleotide Binding Oligomerization Domain Containing 2 | 10 |
| MAVS | Mitochondrial Antiviral Signaling Protein | 9 |
| ATG16L1 | Autophagy Related 16 Like 1 | 12 |
| IFIH1 | Interferon Induced with Helicase C Domain 1 | 9 |
| ATG5 | Autophagy Related 5 | 12 |
| TBK1 | TANK Binding Kinase 1 | 11 |
| CARD9 | Caspase Recruitment Domain Family Member 9 | 8 |
| VWF | von Willebrand factor | 8 |
