# Supplementary material for: Identification of key genes for hypertrophic cardiomyopathy using integrated network analysis of differential lncRNA and gene expression
Source: Front Cardiovasc Med. 2022 Aug 4;9:946229. doi: 10.3389/fcvm.2022.946229 (PMC9386162; doi:10.3389/fcvm.2022.946229)
Supplement: Supplementary file 4 [file Table_4.docx]

**Supplementary table 4: Clinical characteristics of HCM patients and the controls.**

|  | control (n=4) | HCM(n=4) |
| --- | --- | --- |
| Age (years) | 41.50±13.28 | 45.25±10.37 |
| Sex, male (%) | 2 (50%) | 2 (50%) |
| LVEDD (mm) | 43.75±5.44 | 42.75±5.91 |
| LVEF (%) | 66.75±4.27 | 68.50±3.11 |
| IVST (mm) | 8.25±2.22 | 21.45±2.03 |
| LVPW (mm) | 7.20±1.83 | 14.00±3.92 |

Abbreviation: LVEDD, Left ventricular end-diastolic diameter; LVEF, Left ventricular ejection fraction; IVST, Interventricular septal thickness; LVPW, Left ventricular posterior wall.
