# Supplementary material for: Identification of key genes for hypertrophic cardiomyopathy using integrated network analysis of differential lncRNA and gene expression
Source: Front Cardiovasc Med. 2022 Aug 4;9:946229. doi: 10.3389/fcvm.2022.946229 (PMC9386162; doi:10.3389/fcvm.2022.946229)
Supplement: Supplementary file 5 [file Table_5.docx]

**Supplementary table 5: Primer sequences for real-time PCR.**

| Primer name | sequence |
| --- | --- |
| LA16c-312E8.2-forward | AACCAAGAGCAACGCCTGATACG |
| LA16c-312E8.2-reverse | CAGCACGGAGATCCTTGTGAAGTC |
| RP5-1160K1.3-forward | CCCAGGCTGAGGCAGGAGAATC |
| RP5-1160K1.3-reverse | GGCTGGAATTCAGTGGTGCGATC |
| MIR22HG-forward | CCATACATTGCGTGTGGGAG |
| MIR22HG-reverse | TTCGTAGGTCAAATG ACATGGAG |
| LINC00324-forward | TGTGGATGACAGTGTTCGGG |
| LINC00324-reverse | ACGCTGACCAGAAACCGTAG |
| SNHG12-forward | CCTTGCCTCTTAATTAAGGGTGG |
| SNHG12-reverse | CTTGCCTTCTGCTTCCCATAG |
| GAPDH-forward | GGGAGCCAAAAGGGTCAT |
| GAPDH -reverse | GAGTCCTTCCACGATACCAA |
